# Supplementary material for: The day/night difference in the circadian clock's response to acute lipopolysaccharide and the rhythmic Stat3 expression in the rat suprachiasmatic nucleus
Source: PLoS One. 2018 Sep 28;13(9):e0199405. doi: 10.1371/journal.pone.0199405 (PMC6161871; doi:10.1371/journal.pone.0199405)
Supplement: S1 Fig — Adult rats were injected with LPS (1 mg/kg) either during the day, at ZT6, or at night, at ZT15, and sampled 2 h, 5 h, 8 h and 24 h later. The levels of Per1 (A), Per2 (B) and Nr1d1 (C) mRNAs were assessed as the intensity of the hybridisation signal. Each column represents the mean of four values ± SEM. # P: Values of multiple t-tests with the Sidak-Bonferroni post-hoc test. (DOCX) [file pone.0199405.s001.docx]

S1 Fig: Effect of acute systemic LPS administration on clock gene expression in rat SCN.


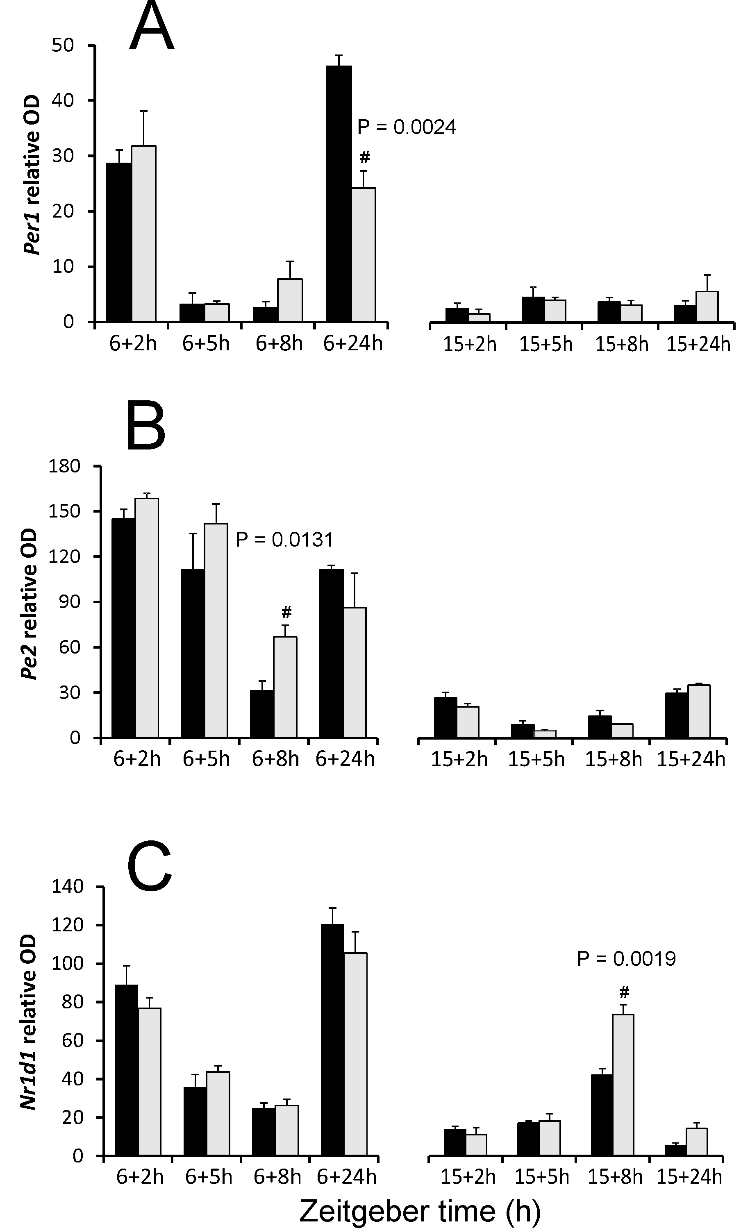


Adult rats were injected with LPS (1 mg/kg) either during the day, at ZT6, or at night, at ZT15, and sampled 2 h, 5 h, 8 h and 24 h later. The levels of *Per1* (A), *Per2* (B) and *Nr1d1* (C) mRNAs were assessed as the intensity of the hybridisation signal. Each column represents the mean of four values ± SEM. # P: Values of multiple t-tests with the Sidak-Bonferroni post-hoc test.
