# Supplementary material for: The day/night difference in the circadian clock's response to acute lipopolysaccharide and the rhythmic Stat3 expression in the rat suprachiasmatic nucleus
Source: PLoS One. 2018 Sep 28;13(9):e0199405. doi: 10.1371/journal.pone.0199405 (PMC6161871; doi:10.1371/journal.pone.0199405)
Supplement: S2 Fig — Effect of acute systemic LPS administration on pERK1/2 (A, B) and pGSK3β (C, D) phosphorylation within rat SCN. Adult rats were injected with LPS (1 mg/kg) either during the day, at ZT6, or at night, at ZT15, and sampled 2 h, 5 h, 8 h and 24 h later (grey columns) (controls; black columns). Levels of pERK1/2 (A, B) and pGSK3β (C, D) were assessed separately for the ventrolateral (A, C) and dorsomedial (B, D) SCN. Each column represents the mean of four values ± SEM. # P: Value of multiple t-tests with the Sidak-Bonferroni post-hoc test. (DOCX) [file pone.0199405.s002.docx]

S2 Fig: Effect of acute systemic LPS administration on pERK1/2 (A, B) and pGSK3β (C, D) phosphorylation within rat SCN.


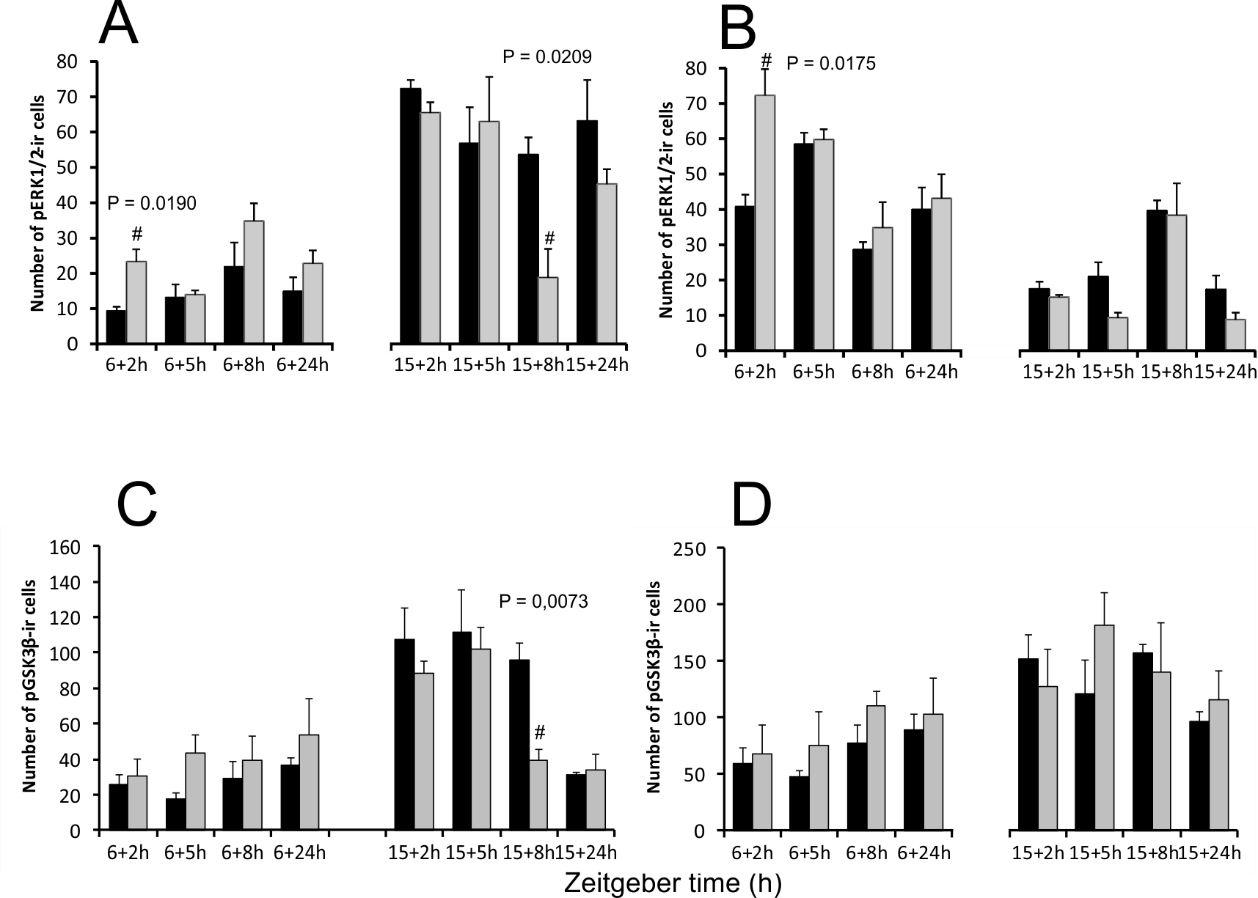


Adult rats were injected with LPS (1 mg/kg) either during the day, at ZT6, or at night, at ZT15, and sampled 2 h, 5 h, 8 h and 24 h later (grey columns) (controls; black columns). Levels of pERK1/2 (A, B) and pGSK3β (C, D) were assessed separately for the ventrolateral (A, C) and dorsomedial (B, D) SCN. Each column represents the mean of four values ± SEM. # P: Value of multiple t-tests with the Sidak-Bonferroni post-hoc test.
